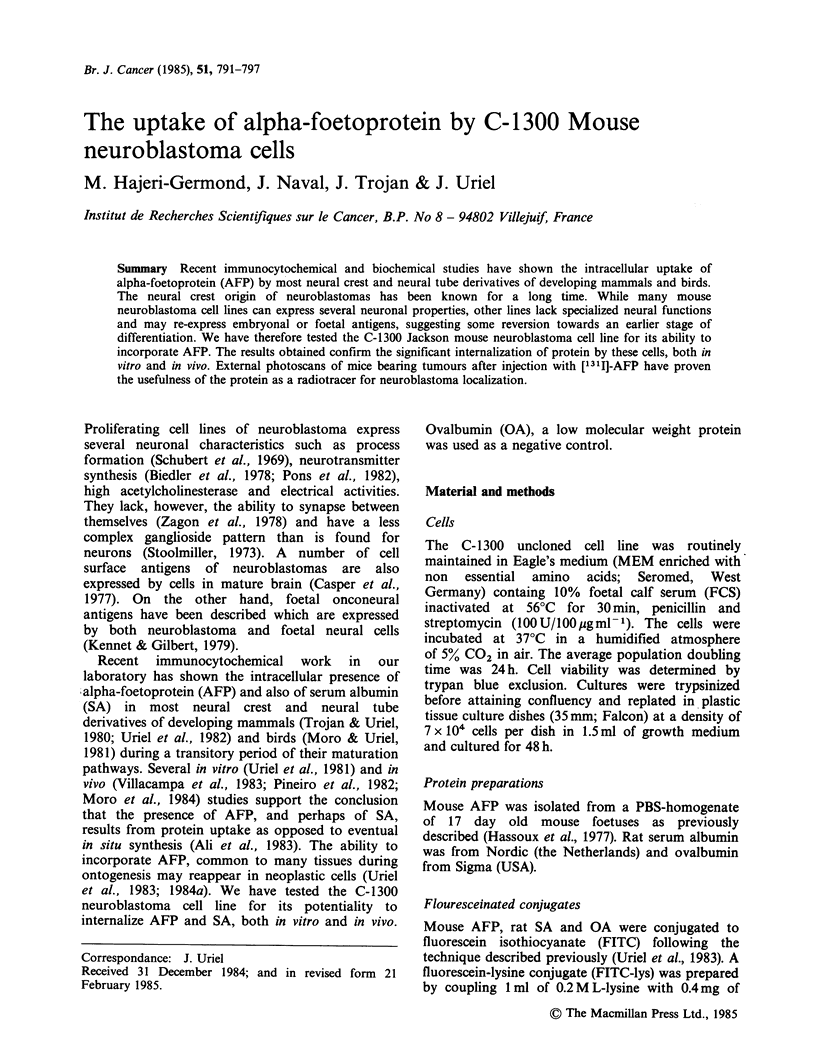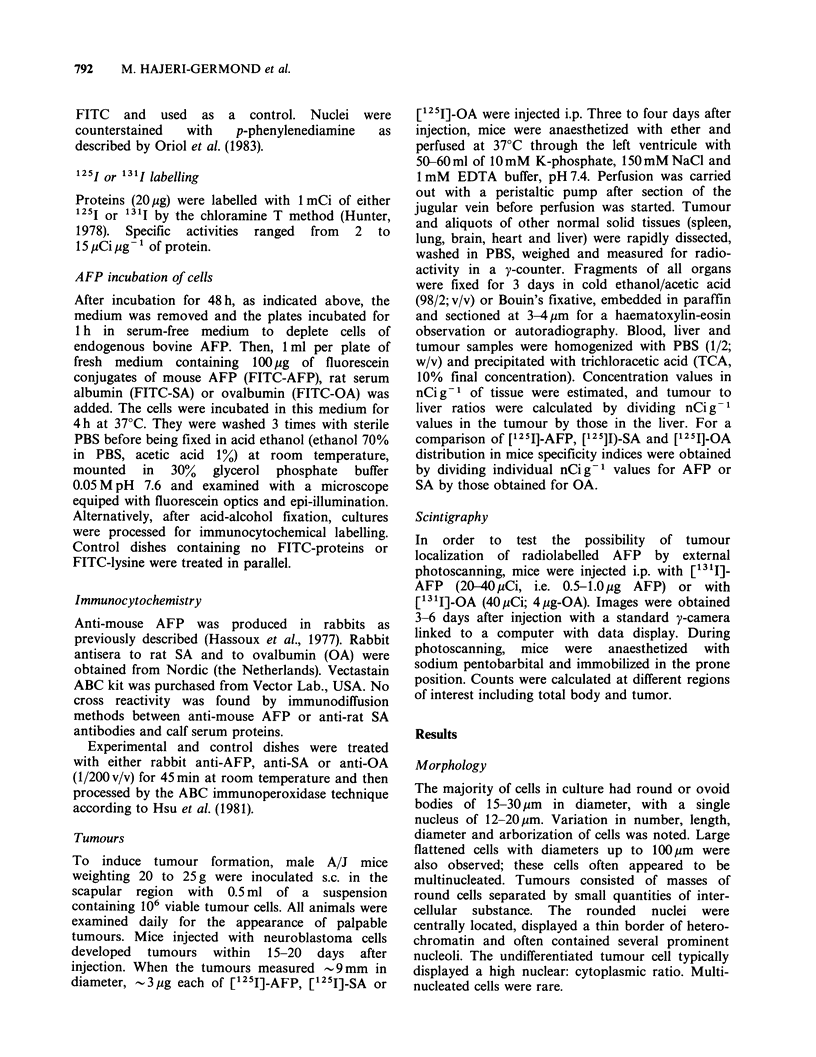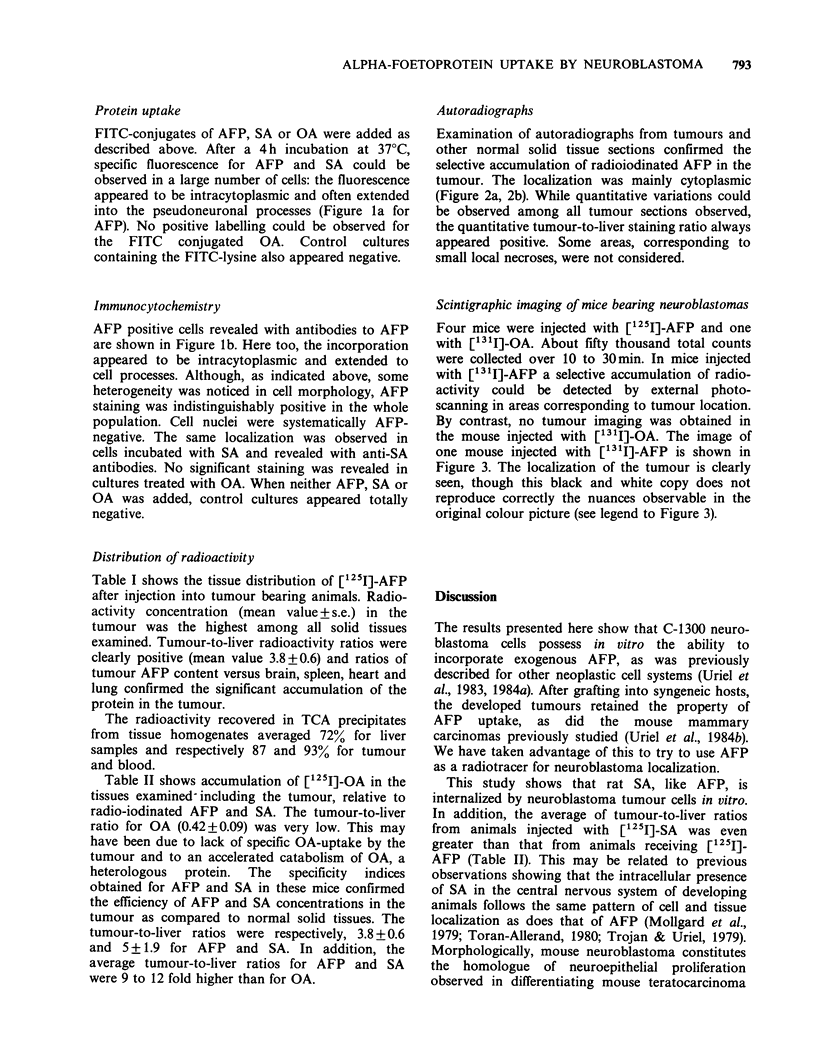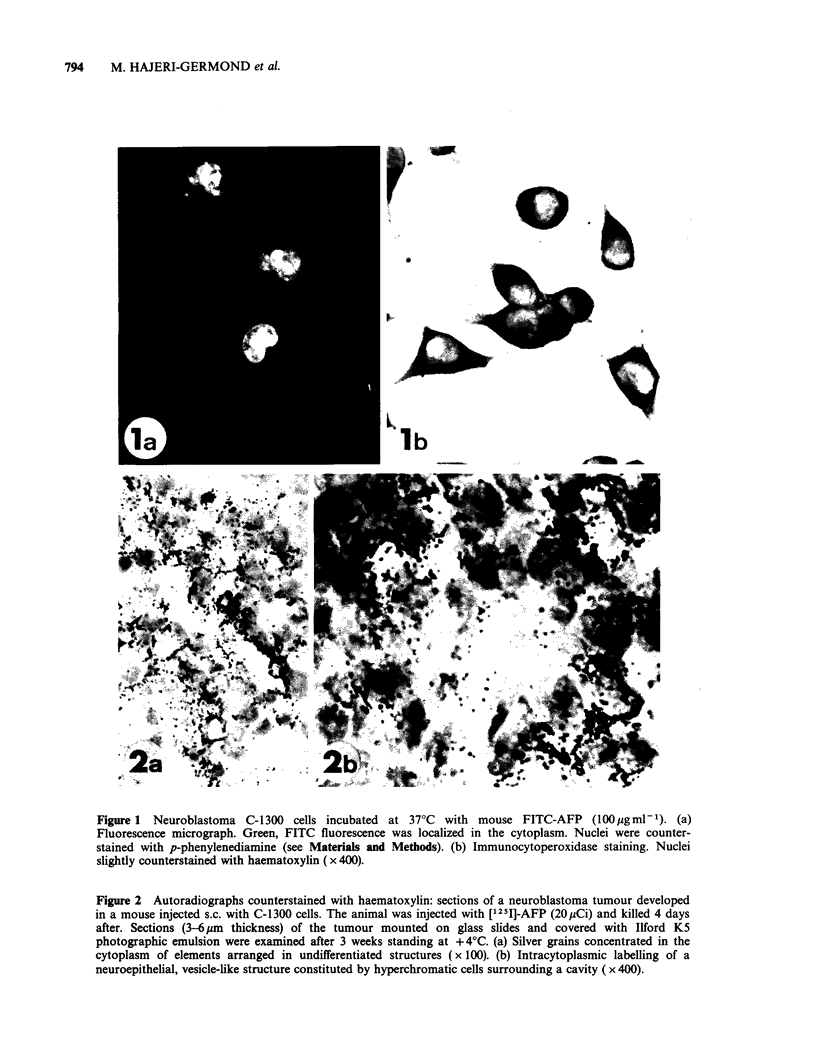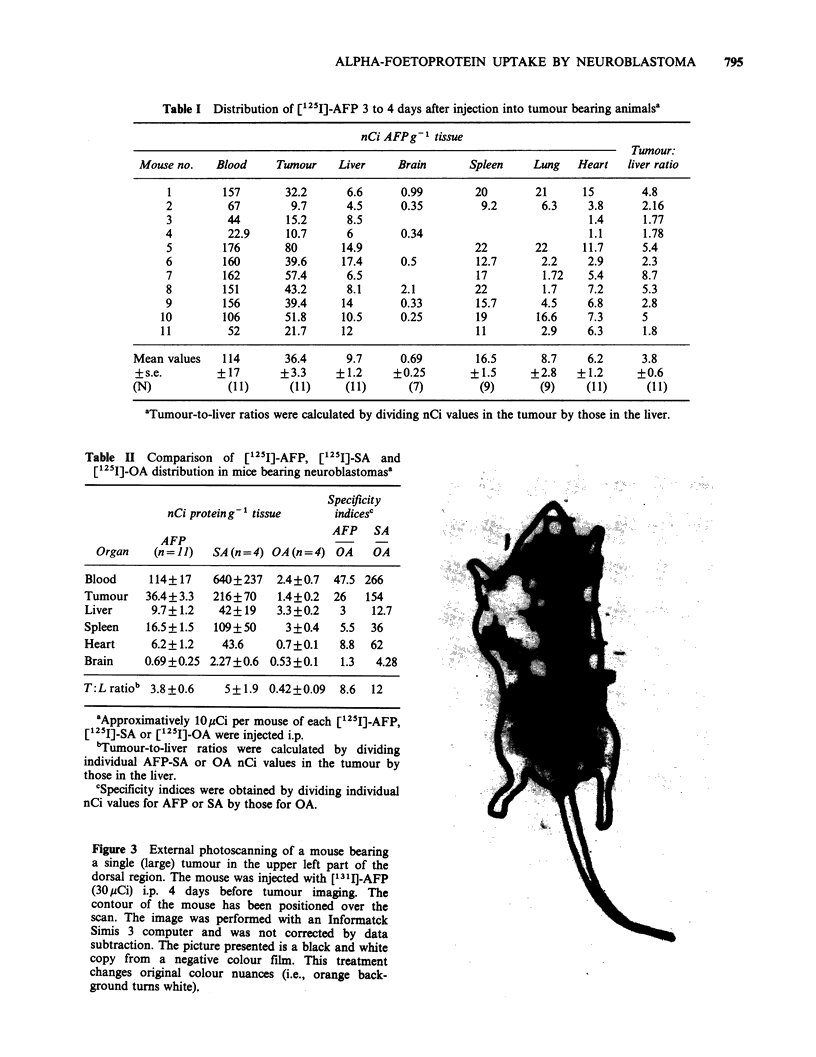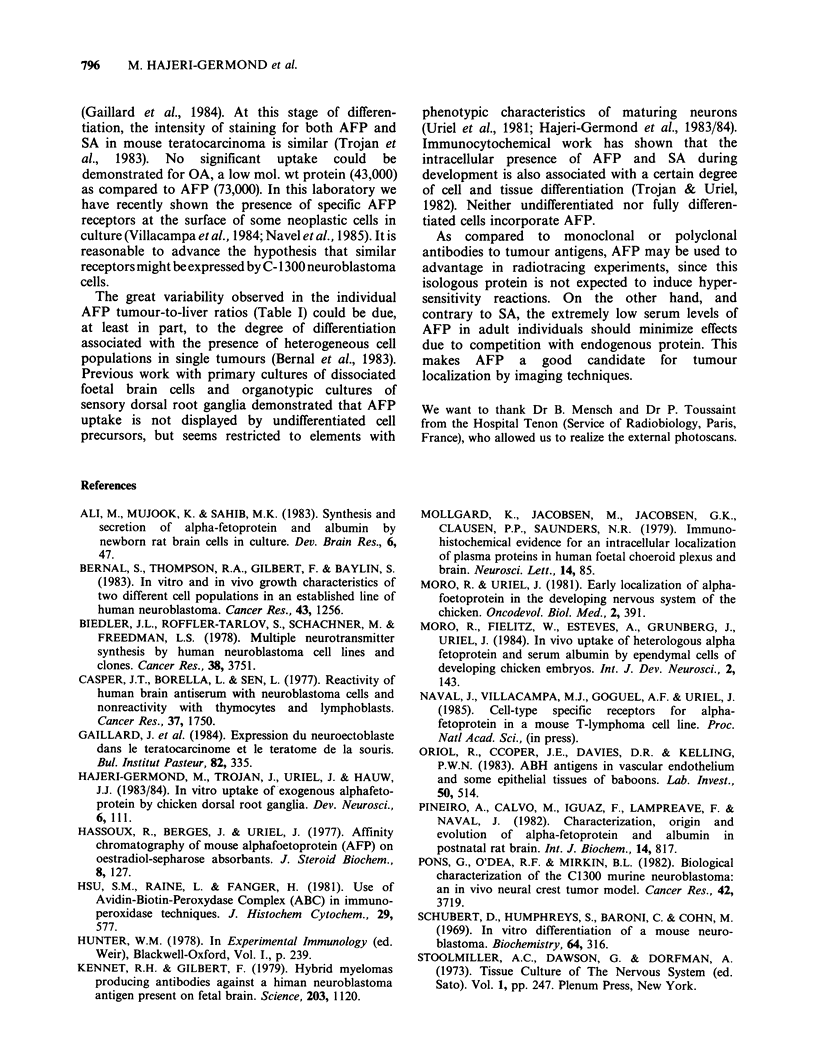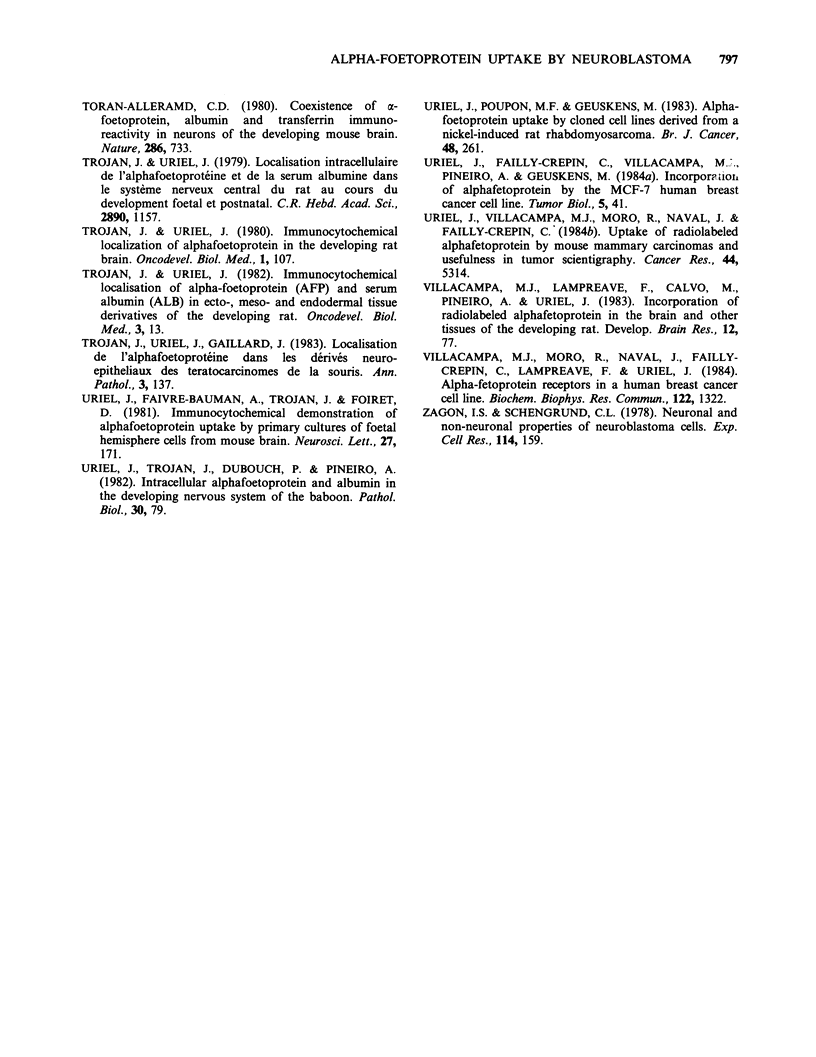# The uptake of alpha-foetoprotein by C-1300 mouse neuroblastoma cells.

**DOI:** 10.1038/bjc.1985.123

**Published:** 1985-06

**Authors:** M. Hajeri-Germond, J. Naval, J. Trojan, J. Uriel

## Abstract

**Images:**


*Br. J. Cancer* (1985), **51**, 791–797

### The uptake of alpha-foetoprotein by C-1300 Mouse neuroblastoma cells

M. Hajeri-Germond, J. Naval, J. Trojan & J. Uriel

*Institut de Recherches Scientifiques sur le Cancer, B.P. No 8 – 94802 Villejuif, France*

**Summary** Recent immunocytochemical and biochemical studies have shown the intracellular uptake of alpha-foetoprotein (AFP) by most neural crest and neural tube derivatives of developing mammals and birds. The neural crest origin of neuroblastomas has been known for a long time. While many mouse neuroblastoma cell lines can express several neuronal properties, other lines lack specialized neural functions and may re-express embryonal or foetal antigens, suggesting some reversion towards an earlier stage of differentiation. We have therefore tested the C-1300 Jackson mouse neuroblastoma cell line for its ability to incorporate AFP. The results obtained confirm the significant internalization of protein by these cells, both *in vitro* and *in vivo*. External photoscans of mice bearing tumours after injection with [$^{131}$I]-AFP have proven the usefulness of the protein as a radiotracer for neuroblastoma localization.

Proliferating cell lines of neuroblastoma express several neuronal characteristics such as process formation (Schubert *et al.*, 1969), neurotransmitter synthesis (Biedler *et al.*, 1978; Pons *et al.*, 1982), high acetylcholinesterase and electrical activities. They lack, however, the ability to synapse between themselves (Zagon *et al.*, 1978) and have a less complex ganglioside pattern than is found for neurons (Stoolmiller, 1973). A number of cell surface antigens of neuroblastomas are also expressed by cells in mature brain (Casper *et al.*, 1977). On the other hand, foetal onconeural antigens have been described which are expressed by both neuroblastoma and foetal neural cells (Kennet & Gilbert, 1979).

Recent immunocytochemical work in our laboratory has shown the intracellular presence of alpha-foetoprotein (AFP) and also of serum albumin (SA) in most neural crest and neural tube derivatives of developing mammals (Trojan & Uriel, 1980; Uriel *et al.*, 1982) and birds (Moro & Uriel, 1981) during a transitory period of their maturation pathways. Several *in vitro* (Uriel *et al.*, 1981) and *in vivo* (Villacampa *et al.*, 1983; Pineiro *et al.*, 1982; Moro *et al.*, 1984) studies support the conclusion that the presence of AFP, and perhaps of SA, results from protein uptake as opposed to eventual *in situ* synthesis (Ali *et al.*, 1983). The ability to incorporate AFP, common to many tissues during ontogenesis may reappear in neoplastic cells (Uriel *et al.*, 1983; 1984*a*). We have tested the C-1300 neuroblastoma cell line for its potentiality to internalize AFP and SA, both *in vitro* and *in vivo*. Ovalbumin (OA), a low molecular weight protein was used as a negative control.

Correspondance: J. Uriel
Received 31 December 1984; and in revised form 21 February 1985.

### Material and methods

### *Cells*

The C-1300 uncloned cell line was routinely maintained in Eagle's medium (MEM enriched with non essential amino acids; Seromed, West Germany) containg 10% foetal calf serum (FCS) inactivated at 56°C for 30 min, penicillin and streptomycin (100 U/100 $\mu$g ml$^{-1}$). The cells were incubated at 37°C in a humidified atmosphere of 5% $CO_2$ in air. The average population doubling time was 24 h. Cell viability was determined by trypan blue exclusion. Cultures were trypsinized before attaining confluency and replated in plastic tissue culture dishes (35 mm; Falcon) at a density of $7 \times 10^4$ cells per dish in 1.5 ml of growth medium and cultured for 48 h.

### *Protein preparations*

Mouse AFP was isolated from a PBS-homogenate of 17 day old mouse foetuses as previously described (Hassoux *et al.*, 1977). Rat serum albumin was from Nordic (the Netherlands) and ovalbumin from Sigma (USA).

### *Flouresceinated conjugates*

Mouse AFP, rat SA and OA were conjugated to fluorescein isothiocyanate (FITC) following the technique described previously (Uriel *et al.*, 1983). A fluorescein-lysine conjugate (FITC-lys) was prepared by coupling 1 ml of 0.2 M L-lysine with 0.4 mg of

© The Macmillan Press Ltd., 1985

FITC and used as a control. Nuclei were counterstained with *p*-phenylenediamine as described by Oriol *et al.* (1983).

### $^{125}I$ or $^{131}I$ labelling

Proteins (20 $\mu$g) were labelled with 1 mCi of either $^{125}$I or $^{131}$I by the chloramine T method (Hunter, 1978). Specific activities ranged from 2 to 15 $\mu$Ci $\mu g^{-1}$ of protein.

### AFP incubation of cells

After incubation for 48 h, as indicated above, the medium was removed and the plates incubated for 1 h in serum-free medium to deplete cells of endogenous bovine AFP. Then, 1 ml per plate of fresh medium containing 100 $\mu$g of fluorescein conjugates of mouse AFP (FITC-AFP), rat serum albumin (FITC-SA) or ovalbumin (FITC-OA) was added. The cells were incubated in this medium for 4 h at 37°C. They were washed 3 times with sterile PBS before being fixed in acid ethanol (ethanol 70% in PBS, acetic acid 1%) at room temperature, mounted in 30% glycerol phosphate buffer 0.05 M pH 7.6 and examined with a microscope equiped with fluorescein optics and epi-illumination. Alternatively, after acid-alcohol fixation, cultures were processed for immunocytochemical labelling. Control dishes containing no FITC-proteins or FITC-lysine were treated in parallel.

### Immunocytochemistry

Anti-mouse AFP was produced in rabbits as previously described (Hassoux *et al.*, 1977). Rabbit antisera to rat SA and to ovalbumin (OA) were obtained from Nordic (the Netherlands). Vectastain ABC kit was purchased from Vector Lab., USA. No cross reactivity was found by immunodiffusion methods between anti-mouse AFP or anti-rat SA antibodies and calf serum proteins.

Experimental and control dishes were treated with either rabbit anti-AFP, anti-SA or anti-OA (1/200 v/v) for 45 min at room temperature and then processed by the ABC immunoperoxidase technique according to Hsu *et al.* (1981).

### Tumours

To induce tumour formation, male A/J mice weighting 20 to 25 g were inoculated s.c. in the scapular region with 0.5 ml of a suspension containing $10^6$ viable tumour cells. All animals were examined daily for the appearance of palpable tumours. Mice injected with neuroblastoma cells developed tumours within 15–20 days after injection. When the tumours measured ~9 mm in diameter, ~3 $\mu$g each of [$^{125}$I]-AFP, [$^{125}$I]-SA or [$^{125}$I]-OA were injected i.p. Three to four days after injection, mice were anaesthetized with ether and perfused at 37°C through the left ventricule with 50–60 ml of 10 mM K-phosphate, 150 mM NaCl and 1 mM EDTA buffer, pH 7.4. Perfusion was carried out with a peristaltic pump after section of the jugular vein before perfusion was started. Tumour and aliquots of other normal solid tissues (spleen, lung, brain, heart and liver) were rapidly dissected, washed in PBS, weighed and measured for radioactivity in a $\gamma$-counter. Fragments of all organs were fixed for 3 days in cold ethanol/acetic acid (98/2; v/v) or Bouin's fixative, embedded in paraffin and sectioned at 3–4 $\mu$m for a haematoxylin-eosin observation or autoradiography. Blood, liver and tumour samples were homogenized with PBS (1/2; w/v) and precipitated with trichloracetic acid (TCA, 10% final concentration). Concentration values in nCi $g^{-1}$ of tissue were estimated, and tumour to liver ratios were calculated by dividing nCi $g^{-1}$ values in the tumour by those in the liver. For a comparison of [$^{125}$I]-AFP, [$^{125}$]I)-SA and [$^{125}$I]-OA distribution in mice specificity indices were obtained by dividing individual nCi $g^{-1}$ values for AFP or SA by those obtained for OA.

### Scintigraphy

In order to test the possibility of tumour localization of radiolabelled AFP by external photoscanning, mice were injected i.p. with [$^{131}$I]-AFP (20–40 $\mu$Ci, i.e. 0.5–1.0 $\mu$g AFP) or with [$^{131}$I]-OA (40 $\mu$Ci; 4 $\mu$g-OA). Images were obtained 3–6 days after injection with a standard $\gamma$-camera linked to a computer with data display. During photoscanning, mice were anaesthetized with sodium pentobarbital and immobilized in the prone position. Counts were calculated at different regions of interest including total body and tumor.

### Results

### Morphology

The majority of cells in culture had round or ovoid bodies of 15–30 $\mu$m in diameter, with a single nucleus of 12–20 $\mu$m. Variation in number, length, diameter and arborization of cells was noted. Large flattened cells with diameters up to 100 $\mu$m were also observed; these cells often appeared to be multinucleated. Tumours consisted of masses of round cells separated by small quantities of intercellular substance. The rounded nuclei were centrally located, displayed a thin border of heterochromatin and often contained several prominent nucleoli. The undifferentiated tumour cell typically displayed a high nuclear: cytoplasmic ratio. Multinucleated cells were rare.

*Protein uptake*

FITC-conjugates of AFP, SA or OA were added as described above. After a 4 h incubation at 37°C, specific fluorescence for AFP and SA could be observed in a large number of cells: the fluorescence appeared to be intracytoplasmic and often extended into the pseudoneuronal processes (Figure 1a for AFP). No positive labelling could be observed for the FITC conjugated OA. Control cultures containing the FITC-lysine also appeared negative.

*Immunocytochemistry*

AFP positive cells revealed with antibodies to AFP are shown in Figure 1b. Here too, the incorporation appeared to be intracytoplasmic and extended to cell processes. Although, as indicated above, some heterogeneity was noticed in cell morphology, AFP staining was indistinguishably positive in the whole population. Cell nuclei were systematically AFP-negative. The same localization was observed in cells incubated with SA and revealed with anti-SA antibodies. No significant staining was revealed in cultures treated with OA. When neither AFP, SA or OA was added, control cultures appeared totally negative.

*Distribution of radioactivity*

Table I shows the tissue distribution of [$^{125}$I]-AFP after injection into tumour bearing animals. Radioactivity concentration (mean value ± s.e.) in the tumour was the highest among all solid tissues examined. Tumour-to-liver radioactivity ratios were clearly positive (mean value 3.8 ± 0.6) and ratios of tumour AFP content versus brain, spleen, heart and lung confirmed the significant accumulation of the protein in the tumour.

The radioactivity recovered in TCA precipitates from tissue homogenates averaged 72% for liver samples and respectively 87 and 93% for tumour and blood.

Table II shows accumulation of [$^{125}$I]-OA in the tissues examined including the tumour, relative to radio-iodinated AFP and SA. The tumour-to-liver ratio for OA (0.42 ± 0.09) was very low. This may have been due to lack of specific OA-uptake by the tumour and to an accelerated catabolism of OA, a heterologous protein. The specificity indices obtained for AFP and SA in these mice confirmed the efficiency of AFP and SA concentrations in the tumour as compared to normal solid tissues. The tumour-to-liver ratios were respectively, 3.8 ± 0.6 and 5 ± 1.9 for AFP and SA. In addition, the average tumour-to-liver ratios for AFP and SA were 9 to 12 fold higher than for OA.

*Autoradiographs*

Examination of autoradiographs from tumours and other normal solid tissue sections confirmed the selective accumulation of radioiodinated AFP in the tumour. The localization was mainly cytoplasmic (Figure 2a, 2b). While quantitative variations could be observed among all tumour sections observed, the quantitative tumour-to-liver staining ratio always appeared positive. Some areas, corresponding to small local necroses, were not considered.

*Scintigraphic imaging of mice bearing neuroblastomas*

Four mice were injected with [$^{125}$I]-AFP and one with [$^{131}$I]-OA. About fifty thousand total counts were collected over 10 to 30 min. In mice injected with [$^{131}$I]-AFP a selective accumulation of radioactivity could be detected by external photoscanning in areas corresponding to tumour location. By contrast, no tumour imaging was obtained in the mouse injected with [$^{131}$I]-OA. The image of one mouse injected with [$^{131}$I]-AFP is shown in Figure 3. The localization of the tumour is clearly seen, though this black and white copy does not reproduce correctly the nuances observable in the original colour picture (see legend to Figure 3).

### Discussion

The results presented here show that C-1300 neuroblastoma cells possess *in vitro* the ability to incorporate exogenous AFP, as was previously described for other neoplastic cell systems (Uriel *et al.*, 1983, 1984*a*). After grafting into syngeneic hosts, the developed tumours retained the property of AFP uptake, as did the mouse mammary carcinomas previously studied (Uriel *et al.*, 1984*b*). We have taken advantage of this to try to use AFP as a radiotracer for neuroblastoma localization.

This study shows that rat SA, like AFP, is internalized by neuroblastoma tumour cells *in vitro*. In addition, the average of tumour-to-liver ratios from animals injected with [$^{125}$I]-SA was even greater than that from animals receiving [$^{125}$I]-AFP (Table II). This may be related to previous observations showing that the intracellular presence of SA in the central nervous system of developing animals follows the same pattern of cell and tissue localization as does that of AFP (Mollgard *et al.*, 1979; Toran-Allerand, 1980; Trojan & Uriel, 1979). Morphologically, mouse neuroblastoma constitutes the homologue of neuroepithelial proliferation observed in differentiating mouse teratocarcinoma

**Figure 1** Neuroblastoma C-1300 cells incubated at 37°C with mouse FITC-AFP (100 $\mu$g ml$^{-1}$). (a) Fluorescence micrograph. Green, FITC fluorescence was localized in the cytoplasm. Nuclei were counterstained with *p*-phenylenediamine (see **Materials and Methods**). (b) Immunocytoperoxidase staining. Nuclei slightly counterstained with haematoxylin ( $\times$ 400).

**Figure 2** Autoradiographs counterstained with haematoxylin: sections of a neuroblastoma tumour developed in a mouse injected s.c. with C-1300 cells. The animal was injected with [$^{125}$I]-AFP (20 $\mu$Ci) and killed 4 days after. Sections (3–6 $\mu$m thickness) of the tumour mounted on glass slides and covered with Ilford K5 photographic emulsion were examined after 3 weeks standing at +4°C. (a) Silver grains concentrated in the cytoplasm of elements arranged in undifferentiated structures ( $\times$ 100). (b) Intracytoplasmic labelling of a neuroepithelial, vesicle-like structure constituted by hyperchromatic cells surrounding a cavity ( $\times$ 400).

**Table I** Distribution of [$^{125}$I]-AFP 3 to 4 days after injection into tumour bearing animals[a]

| | *nCi AFP g$^{-1}$ tissue* | | | | | | | |
|---|---|---|---|---|---|---|---|---|
| *Mouse no.* | *Blood* | *Tumour* | *Liver* | *Brain* | *Spleen* | *Lung* | *Heart* | *Tumour: liver ratio* |
| 1 | 157 | 32.2 | 6.6 | 0.99 | 20 | 21 | 15 | 4.8 |
| 2 | 67 | 9.7 | 4.5 | 0.35 | 9.2 | 6.3 | 3.8 | 2.16 |
| 3 | 44 | 15.2 | 8.5 | | | | 1.4 | 1.77 |
| 4 | 22.9 | 10.7 | 6 | 0.34 | | | 1.1 | 1.78 |
| 5 | 176 | 80 | 14.9 | | 22 | 22 | 11.7 | 5.4 |
| 6 | 160 | 39.6 | 17.4 | 0.5 | 12.7 | 2.2 | 2.9 | 2.3 |
| 7 | 162 | 57.4 | 6.5 | | 17 | 1.72 | 5.4 | 8.7 |
| 8 | 151 | 43.2 | 8.1 | 2.1 | 22 | 1.7 | 7.2 | 5.3 |
| 9 | 156 | 39.4 | 14 | 0.33 | 15.7 | 4.5 | 6.8 | 2.8 |
| 10 | 106 | 51.8 | 10.5 | 0.25 | 19 | 16.6 | 7.3 | 5 |
| 11 | 52 | 21.7 | 12 | | 11 | 2.9 | 6.3 | 1.8 |
| Mean values | 114 | 36.4 | 9.7 | 0.69 | 16.5 | 8.7 | 6.2 | 3.8 |
| ±s.e. | ±17 | ±3.3 | ±1.2 | ±0.25 | ±1.5 | ±2.8 | ±1.2 | ±0.6 |
| (N) | (11) | (11) | (11) | (7) | (9) | (9) | (11) | (11) |

[a]Tumour-to-liver ratios were calculated by dividing nCi values in the tumour by those in the liver.

**Table II** Comparison of [$^{125}$I]-AFP, [$^{125}$I]-SA and [$^{125}$I]-OA distribution in mice bearing neuroblastomas[a]

| | *nCi protein g$^{-1}$ tissue* | | | *Specificity indices*[c] | |
|---|---|---|---|---|---|
| *Organ* | *AFP (n=11)* | *SA (n=4)* | *OA (n=4)* | *AFP/OA* | *SA/OA* |
| Blood | 114±17 | 640±237 | 2.4±0.7 | 47.5 | 266 |
| Tumour | 36.4±3.3 | 216±70 | 1.4±0.2 | 26 | 154 |
| Liver | 9.7±1.2 | 42±19 | 3.3±0.2 | 3 | 12.7 |
| Spleen | 16.5±1.5 | 109±50 | 3±0.4 | 5.5 | 36 |
| Heart | 6.2±1.2 | 43.6 | 0.7±0.1 | 8.8 | 62 |
| Brain | 0.69±0.25 | 2.27±0.6 | 0.53±0.1 | 1.3 | 4.28 |
| *T:L* ratio[b] | 3.8±0.6 | 5±1.9 | 0.42±0.09 | 8.6 | 12 |

[a]Approximatively 10 $\mu$Ci per mouse of each [$^{125}$I]-AFP, [$^{125}$I]-SA or [$^{125}$I]-OA were injected i.p.

[b]Tumour-to-liver ratios were calculated by dividing individual AFP-SA or OA nCi values in the tumour by those in the liver.

[c]Specificity indices were obtained by dividing individual nCi values for AFP or SA by those for OA.

**Figure 3** External photoscanning of a mouse bearing a single (large) tumour in the upper left part of the dorsal region. The mouse was injected with [$^{131}$I]-AFP (30 $\mu$Ci) i.p. 4 days before tumour imaging. The contour of the mouse has been positioned over the scan. The image was performed with an Informatck Simis 3 computer and was not corrected by data subtraction. The picture presented is a black and white copy from a negative colour film. This treatment changes original colour nuances (i.e., orange background turns white),

(Gaillard *et al.*, 1984). At this stage of differentiation, the intensity of staining for both AFP and SA in mouse teratocarcinoma is similar (Trojan *et al.*, 1983). No significant uptake could be demonstrated for OA, a low mol. wt protein (43,000) as compared to AFP (73,000). In this laboratory we have recently shown the presence of specific AFP receptors at the surface of some neoplastic cells in culture (Villacampa *et al.*, 1984; Navel *et al.*, 1985). It is reasonable to advance the hypothesis that similar receptors might be expressed by C-1300 neuroblastoma cells.

The great variability observed in the individual AFP tumour-to-liver ratios (Table I) could be due, at least in part, to the degree of differentiation associated with the presence of heterogeneous cell populations in single tumours (Bernal *et al.*, 1983). Previous work with primary cultures of dissociated foetal brain cells and organotypic cultures of sensory dorsal root ganglia demonstrated that AFP uptake is not displayed by undifferentiated cell precursors, but seems restricted to elements with phenotypic characteristics of maturing neurons (Uriel *et al.*, 1981; Hajeri-Germond *et al.*, 1983/84). Immunocytochemical work has shown that the intracellular presence of AFP and SA during development is also associated with a certain degree of cell and tissue differentiation (Trojan & Uriel, 1982). Neither undifferentiated nor fully differentiated cells incorporate AFP.

As compared to monoclonal or polyclonal antibodies to tumour antigens, AFP may be used to advantage in radiotracing experiments, since this isologous protein is not expected to induce hypersensitivity reactions. On the other hand, and contrary to SA, the extremely low serum levels of AFP in adult individuals should minimize effects due to competition with endogenous protein. This makes AFP a good candidate for tumour localization by imaging techniques.

We want to thank Dr B. Mensch and Dr P. Toussaint from the Hospital Tenon (Service of Radiobiology, Paris, France), who allowed us to realize the external photoscans.

### References

ALI, M., MUJOOK, K. & SAHIB, M.K. (1983). Synthesis and secretion of alpha-fetoprotein and albumin by newborn rat brain cells in culture. *Dev. Brain Res.*, **6**, 47.

BERNAL, S., THOMPSON, R.A., GILBERT, F. & BAYLIN, S. (1983). In vitro and in vivo growth characteristics of two different cell populations in an established line of human neuroblastoma. *Cancer Res.*, **43**, 1256.

BIEDLER, J.L., ROFFLER-TARLOV, S., SCHACHNER, M. & FREEDMAN, L.S. (1978). Multiple neurotransmitter synthesis by human neuroblastoma cell lines and clones. *Cancer Res.*, **38**, 3751.

CASPER, J.T., BORELLA, L. & SEN, L. (1977). Reactivity of human brain antiserum with neuroblastoma cells and nonreactivity with thymocytes and lymphoblasts. *Cancer Res.*, **37**, 1750.

GAILLARD, J. *et al.* (1984). Expression du neuroectoblaste dans le teratocarcinome et le teratome de la souris. *Bul. Institut Pasteur*, **82**, 335.

HAJERI-GERMOND, M., TROJAN, J., URIEL, J. & HAUW, J.J. (1983/84). In vitro uptake of exogenous alphafetoprotein by chicken dorsal root ganglia. *Dev. Neurosci.*, **6**, 111.

HASSOUX, R., BERGES, J. & URIEL, J. (1977). Affinity chromatography of mouse alphafoetoprotein (AFP) on oestradiol-sepharose absorbants. *J. Steroid Biochem.*, **8**, 127.

HSU, S.M., RAINE, L. & FANGER, H. (1981). Use of Avidin-Biotin-Peroxydase Complex (ABC) in immunoperoxidase techniques. *J. Histochem Cytochem.*, **29**, 577.

HUNTER, W.M. (1978). In *Experimental Immunology* (ed. Weir), Blackwell-Oxford, Vol. I., p. 239.

KENNET, R.H. & GILBERT, F. (1979). Hybrid myelomas producing antibodies against a himan neuroblastoma antigen present on fetal brain. *Science*, **203**, 1120.

MOLLGARD, K., JACOBSEN, M., JACOBSEN, G.K., CLAUSEN, P.P., SAUNDERS, N.R. (1979). Immunohistochemical evidence for an intracellular localization of plasma proteins in human foetal choeroid plexus and brain. *Neurosci. Lett.*, **14**, 85.

MORO, R. & URIEL, J. (1981). Early localization of alpha-foetoprotein in the developing nervous system of the chicken. *Oncodevol. Biol. Med.*, **2**, 391.

MORO, R., FIELITZ, W., ESTEVES, A., GRUNBERG, J., URIEL, J. (1984). In vivo uptake of heterologous alpha fetoprotein and serum albumin by ependymal cells of developing chicken embryos. *Int. J. Dev. Neurosci.*, **2**, 143.

NAVAL, J., VILLACAMPA, M.J., GOGUEL, A.F. & URIEL, J. (1985). Cell-type specific receptors for alpha-fetoprotein in a mouse T-lymphoma cell line. *Proc. Natl Acad. Sci.*, (in press).

ORIOL, R., CCOPER, J.E., DAVIES, D.R. & KELLING, P.W.N. (1983). ABH antigens in vascular endothelium and some epithelial tissues of baboons. *Lab. Invest.*, **50**, 514.

PINEIRO, A., CALVO, M., IGUAZ, F., LAMPREAVE, F. & NAVAL, J. (1982). Characterization, origin and evolution of alpha-fetoprotein and albumin in postnatal rat brain. *Int. J. Biochem.*, **14**, 817.

PONS, G., O'DEA, R.F. & MIRKIN, B.L. (1982). Biological characterization of the C1300 murine neuroblastoma: an in vivo neural crest tumor model. *Cancer Res.*, **42**, 3719.

SCHUBERT, D., HUMPHREYS, S., BARONI, C. & COHN, M. (1969). In vitro differentiation of a mouse neuroblastoma. *Biochemistry*, **64**, 316.

STOOLMILLER, A.C., DAWSON, G. & DORFMAN, A. (1973). Tissue Culture of The Nervous System (ed. Sato). Vol. **1**, pp. 247. Plenum Press, New York.

TORAN-ALLERAMD, C.D. (1980). Coexistence of α-foetoprotein, albumin and transferrin immunoreactivity in neurons of the developing mouse brain. *Nature*, **286**, 733.

TROJAN, J. & URIEL, J. (1979). Localisation intracellulaire de l'alphafoetoprotéine et de la serum albumine dans le système nerveux central du rat au cours du development foetal et postnatal. *C.R. Hebd. Acad. Sci.*, **2890**, 1157.

TROJAN, J. & URIEL, J. (1980). Immunocytochemical localization of alphafoetoprotein in the developing rat brain. *Oncodevel. Biol. Med.*, **1**, 107.

TROJAN, J. & URIEL, J. (1982). Immunocytochemical localisation of alpha-foetoprotein (AFP) and serum albumin (ALB) in ecto-, meso- and endodermal tissue derivatives of the developing rat. *Oncodevel. Biol. Med.*, **3**, 13.

TROJAN, J., URIEL, J., GAILLARD, J. (1983). Localisation de l'alphafoetoprotéine dans les dérivés neuroepitheliaux des teratocarcinomes de la souris. *Ann. Pathol.*, **3**, 137.

URIEL, J., FAIVRE-BAUMAN, A., TROJAN, J. & FOIRET, D. (1981). Immunocytochemical demonstration of alphafoetoprotein uptake by primary cultures of foetal hemisphere cells from mouse brain. *Neurosci. Lett.*, **27**, 171.

URIEL, J., TROJAN, J., DUBOUCH, P. & PINEIRO, A. (1982). Intracellular alphafoetoprotein and albumin in the developing nervous system of the baboon. *Pathol. Biol.*, **30**, 79.

URIEL, J., POUPON, M.F. & GEUSKENS, M. (1983). Alphafoetoprotein uptake by cloned cell lines derived from a nickel-induced rat rhabdomyosarcoma. *Br. J. Cancer*, **48**, 261.

URIEL, J., FAILLY-CREPIN, C., VILLACAMPA, M.J., PINEIRO, A. & GEUSKENS, M. (1984*a*). Incorporation of alphafetoprotein by the MCF-7 human breast cancer cell line. *Tumor Biol.*, **5**, 41.

URIEL, J., VILLACAMPA, M.J., MORO, R., NAVAL, J. & FAILLY-CREPIN, C. (1984*b*). Uptake of radiolabeled alphafetoprotein by mouse mammary carcinomas and usefulness in tumor scientigraphy. *Cancer Res.*, **44**, 5314.

VILLACAMPA, M.J., LAMPREAVE, F., CALVO, M., PINEIRO, A. & URIEL, J. (1983). Incorporation of radiolabeled alphafetoprotein in the brain and other tissues of the developing rat. Develop. *Brain Res.*, **12**, 77.

VILLACAMPA, M.J., MORO, R., NAVAL, J., FAILLY-CREPIN, C., LAMPREAVE, F. & URIEL, J. (1984). Alpha-fetoprotein receptors in a human breast cancer cell line. *Biochem. Biophys. Res. Commun.*, **122**, 1322.

ZAGON, I.S. & SCHENGRUND, C.L. (1978). Neuronal and non-neuronal properties of neuroblastoma cells. *Exp. Cell Res.*, **114**, 159.